# The impact of ADHD on the health and well-being of ADHD children and their siblings

**DOI:** 10.1007/s00787-016-0841-6

**Published:** 2016-04-01

**Authors:** Tessa Peasgood, Anupam Bhardwaj, Katie Biggs, John E. Brazier, David Coghill, Cindy L. Cooper, David Daley, Cyril De Silva, Val Harpin, Paul Hodgkins, Amulya Nadkarni, Juliana Setyawan, Edmund J. S. Sonuga-Barke

**Affiliations:** 1 School of Health and Related Research (ScHARR), Regent Court, 30 Regent Street, Sheffield, S1 4DA UK; 2 North East London Foundation Trust and University College of London, London, UK; 3 The Department of Psychiatry, The University of Dundee, Dundee, UK; 4 Division of Psychiatry & Applied Psychology, School of Medicine, University of Nottingham, Nottingham, UK; 5 Medway NHS Foundation Trust, Kent, UK; 6 Sheffield Children’s NHS Foundation Trust, Sheffield, UK; 7 Global HEOR and Epidemiology, Shire, 725 Chesterbrook Boulevard, Wayne, PA 19087 USA; 8 Lincolnshire Partnership NHS Foundation Trust, Lincolnshire, UK; 9 Department of Psychology, University of Southampton, Southampton, UK

**Keywords:** ADHD, Children, Siblings, Burden, Well-being, CHU-9D, EQ-5D-Y, Life satisfaction, Sleep, Health-related quality of life, Utility, Impact of ADHD on family outcomes

## Abstract

Childhood attention-deficit/hyperactivity disorder (ADHD) has been associated with reduced health and well-being of patients and their families. The authors undertook a large UK survey-based observational study of the burden associated with childhood ADHD. The impact of ADHD on both the patient (*N* = 476) and their siblings (*N* = 337) on health-related quality of life (HRQoL) and happiness was quantified using multiple standard measures [e.g. child health utility-9D (CHU-9D), EuroQol-5D-Youth]. In the analysis, careful statistical adjustments were made to ensure a like-for-like comparison of ADHD families with two different control groups. We controlled for carers’ ADHD symptoms, their employment and relationship status and siblings’ ADHD symptoms. ADHD was associated with a significant deficit in the patient’s HRQoL (with a CHU-9D score of around 6 % lower). Children with ADHD also have less sleep and were less happy with their family and their lives overall. No consistent decrement to the HRQoL of the siblings was identified across the models, except that related to their own conduct problems. The siblings do, however, report lower happiness with life overall and with their family, even when controlling for the siblings own ADHD symptoms. We also find evidence of elevated bullying between siblings in families with a child with ADHD. Overall, the current results suggest that the reduction in quality of life caused by ADHD is experienced both by the child with ADHD and their siblings.

**Electronic supplementary material:**

The online version of this article (doi:10.1007/s00787-016-0841-6) contains supplementary material, which is available to authorized users.

## Introduction

Attention deficit-hyperactivity disorder (ADHD) is a common childhood onset neurodevelopmental disorder, characterized by developmentally inappropriate levels of inattention and/or hyperactivity and impulsivity that can continue throughout life. A recent systematic review of prevalence rates, using the DSM-IV diagnostic criteria, gave estimates between 5.9 and 7.1 % of children worldwide; with males more likely than females to meet the criteria for an overall diagnosis of ADHD and for each of the three DSM-IV subtypes [1]. Estimates of prevalence in the UK have tended to be slightly lower [2]. Children with ADHD face increased difficulties in school having a higher risk of school expulsion or drop out [3] and academic underperformance [4]. Socially they may struggle with both peer [5] and family relationships [6], and be more at risk of both bullying and being bullied [7]. Children with ADHD may develop significant conduct problems and antisocial behaviours (such as fighting, early substance experimentation and adverse driving outcomes) and increased risk of developing oppositional defiant disorder (ODD) and conduct disorder (CD) [8]. ADHD may affect a child’s emotional well-being in several ways, including feelings of anxiety [9], lower self-esteem [10] poorer psychosocial health [11], and poorer overall quality of life [12–14]. Given the breadth of impact of ADHD, there are likely to be important implications for the well-being of those with whom they spend time, particularly their siblings. Having a sibling with ADHD has been found to impact upon children’s well-being and the quality of their family life [15]. A qualitative study by Kendall [16] found that the impact upon siblings focused on feelings of victimization (from aggressive and annoying acts by their sibling), caretaking (expectations of parents that they befriend and protect their siblings), and feelings of anxiety and sorrow. Siblings describe the constant disruption to family life as “chaotic, conflictual and exhausting” [16, p. 7].

For policy makers there is increasing interest in knowing whether these health and quality of life burdens can be identified using preference based measures of health-related quality of life (HRQoL), like the EuroQol-5D (EQ-5D) [17, 18]. These provide information that can be used to examine the cost-effectiveness of new interventions in terms of cost per quality adjusted life year (QALY) by agencies such as NICE in the UK [19] and related organizations in Australia, Canada, Netherlands and others [20]. Instruments which provide the ‘*Q*’, or utility,^1^ part of the QALY allow health states to be valued on a scale where 0 is equivalent to being dead and 1 is equivalent to full health. There is only minimal evidence within the existing literature on the utility values associated with ADHD-related health states [21–24]. These existing studies used either utility instruments completed by parents as a proxy or values from parent or adult preferences towards ADHD-related vignettes. There are no utility values available that are based on children assessing themselves directly. Furthermore, despite the growing interest in the impact of health conditions on measures of subjective well-being, such as satisfaction with life overall [25], few studies have examined this in relation to ADHD.

The aim of this study was to examine the impact of ADHD on health (particularly as measured by utility) and well-being outcomes for patients themselves and their siblings through comparison with carefully matched control groups. In addressing this we give careful attention to the fact that ADHD tends to cluster in families [26, 27] and explore including controls for both parental and sibling’s own ADHD symptoms.

## Methods

The study obtained ethical approval from the Sheffield Ethics Research Committee, research governance was approved in each research site, and written consent was obtained from all participants.

### Study participants

A large cross-section, observational survey was conducted across 15 ADHD centres/clinics in England and Scotland (Coventry, Derby, Dundee, Durham, Leicester, Lincoln, Medway, Newcastle, Tyne area, North Essex, Nottingham, Rotherham, Sheffield, Southampton, South Staffordshire) from December 2010 to September 2012. Families were invited to participate in the study if they had a child (or children), aged 6–18, with a current diagnosis of ADHD and attending one of the ADHD clinics. The children had all received a clinical diagnosis of ADHD although centres/clinics varied in their diagnostic protocols and instruments used. This sample was representative of a typical UK ADHD-clinic population receiving treatment. The sampling frame covered a wide geographical area and included both specialist mental health services (CAMHS) and specialist paediatric clinics. Data relating to parents or carers and siblings living with the child with ADHD were also collected. Children with a formal diagnosis of CD were excluded to maintain a tight focus within the study on ADHD and remove its confounding effect on the impact of ADHD. The combination of ADHD and CD may be aetiologically distinct from ADHD alone [28].

There were two different control groups. The first control group was taken from wave 1 of the Youth Panel (2009–2010) of ‘Understanding Society: The UK’s Household Longitudinal Survey’ (USoc), a multi-topic household survey in the UK, conducted by the Institute for Social and Economic Research (ISER) [29]. This offered a large sample of 10–15 year olds, although only some of the instruments used in the current study are available in this dataset. A second control group of families was recruited from the South Yorkshire Cohort (SYC)^2^ [30], which enlisted 18,000 patients via GP practices across South Yorkshire. A sample of families from this cohort were sent a request to participate in our study and those who responded positively were sent the full set of questionnaires. Families with a child with a diagnosis of ADHD were excluded. These families completed the same survey instruments as the ADHD-family group.

Five hundred and forty-nine families with a child with ADHD consented to the study. Of these no information was collected on 4 families, and only medical information on the children with ADHD was collected for 90 families. Questionnaire data was collected on 455 ADHD-group families. Of these 394 had 1 child aged 6–18 diagnosed with ADHD, 51 had 2 children diagnosed with ADHD, 8 had 3, 1 had 4 and 1 had 5. Sufficient questionnaire data was collected on 476 of these 529 children (90 %) for them to be included in the analysis. Of the 455 ADHD-group families there were a total of 392 eligible siblings (aged 6–18, living at home, without a diagnosis of ADHD) of which sufficient questionnaire data was collected on 337 (86 %). Data on 123 control families were collected from the SYC. Of these families 61 had 1 child, 51 had 2 children, 10 had 3 children and 1 had 4 children. Some questionnaire data was collected on 196 of these 197 eligible children. Only those children with siblings in the eligible age group were compared to the siblings in the ADHD family group (*N* = 136).

### Instruments

#### Child health utility-9D (CHU-9D)

The CHU-9D [31] is a paediatric generic preference-based measure of HRQoL for children aged 7–17. The CHU-9D has nine attributes: worried, sad, pain, tired, annoyed, schoolwork/homework, sleep, daily routine and able to join in activities, with five response levels for each. The CHU-9D descriptive system was developed from qualitative interviews with school age children.

A set of preference weights has been derived from the application of the standard gamble method from 300 members of the UK adult population [32]. This gives estimates for the importance of a change in one item versus a change in another item and versus extending years of life, as perceived by adults. The tariff generates a score for each CHU-9D health state on a scale on which 0 is equivalent to being dead and 1 represents full health.

#### EuroQol-5D-Youth (EQ-5D-Y)

The EQ-5D-Y [33] is an age-appropriate generic HRQoL instrument, derived from adapting the adult EQ-5D instrument. The EQ-5D-Y instrument comprises five questions dealing with various aspects of physical and emotional health (walking about, washing/dressing, usual activities, pain/discomfort, worried/sad/unhappy), for which the response to each is one of three possible degrees of impairment. There is no recommended value set to derive a utility score from the EQ-5D-Y profile. The EQ-VAS, usually asked alongside the EQ-5D, is a visual analogue scale for recording an individual’s rating for their current health. This is anchored at the bottom at 0 (the worst health you can imagine) and at the top at 100 (the best health you can imagine).

#### Life satisfaction

Single questions were asked about how happy children were with their family and with their life overall to reflect the child’s view of overall well-being and family life. These were taken from Understanding Society^3^ where they had been piloted successfully with children. Each question uses a 1–7 response scale based on ‘smiley’ faces.

#### Bullying

Four questions were asked about how often brothers and sisters perform acts of bullying at home (hit, kick or push you; take your belongings; call you nasty names; and make fun of you), with the response options of never; not much (1–3 times in the last 6 months); quite a lot (more than 4 times in the last 6 months); and a lot (a few times every week). The same questions were asked for how often the child performs those acts towards their siblings. These questions were also taken from Understanding Society.

#### Sleep

Parents/carers reported the typical bed time and getting up time of their children with ADHD, and all children from the SYC-control group.

#### Adult ADHD

The Adult Self Report Scale (ASRS v1.1; [34]) was completed by parents/carers. This is a six-item screener, based on the DSM-IV TR criteria, with five responses for each item. Where an individual has four or more positive responses this was taken as indicating possible adult ADHD.

#### Strengths and Difficulties Questionnaire (SDQ)

Parents completed the SDQ [35], a behavioural screening questionnaire for 3–16-year olds consisting of five sub-scales each with five items. A score of 0–10 is given for each sub-scale (emotional symptoms, conduct problems, hyperactivity, peer problems, and prosocial).^4^ Children can be classified into ‘unlikely’, ‘possible’ and ‘probable’ for each sub-scale, although such classification would usually be based on completion by teachers and children in addition to parents. The SDQ does not provide a full evaluation or diagnosis of ADHD status, however, it has been found to be a good screening measure in a UK community setting [36].

### Statistical methods

Since there were considerable difference in the background characteristics of the ADHD-family group and the control groups (see Table 1) we took measures to ensure our comparisons were carefully controlled. First we used a process called coarsened exact matching (CEM) [37]. Children were allocated to a subgroup based on their gender, age (3 groups), and the highest education attainment of their primary carer (2 groups), all of which are characteristics which are unlikely to have been caused by the child having ADHD. Children were only included in the analysis where a good match could be found for them. The observations were then assigned a weight in proportion to the number of ADHD-family group and control group observations within each subgroup. This matching process created a better covariate balance between the ADHD-family group and the control groups. Any remaining imbalance in observed variables was further controlled for using standard weighted regression models. The more accurate the match, the less emphasis is put on getting the assumptions implicit in the regression models correct (hence it is less sensitive to choices about whether to include interaction or higher order terms, for example) [38]. Due to discarding data that does not have a good match the model does not extrapolate counterfactual outcomes to areas where good information is unavailable. Throughout the matching and the regression adjustment we still relied on an assumption that there were no important *unobservable* differences between the families with a child with ADHD and those without.

**Table 1:** Background characteristics and descriptive data on the children with ADHD, siblings and their controls, prior to matching

	Children with ADHD and their controls				Siblings and their controls			
	ADHD-family group diagnosed children (*N* = 476)	SYC (*N* = 196)	USoc (10–15 years only) (*N* = 4,234)	ADHD-family group diagnosed children (10–15 years only) (*N* = 307)	ADHD-family group siblings (*N* = 337)	SYC (those with appropriate aged siblings) (*N* = 136)	USoc (those with appropriately aged siblings) (*N* = 3,477)	ADHD family group siblings (10–15 years) (*N* = 178)
Age (mean)	11.8 (SD 2.9) Range 6–18	11.8 (SD 2.9) Range 6–18	12.5 (SD 1.7) Range 10–15	12.4 (SD 1.6) Range 10–15	11.8 (SD 3.6) Range 5–18	11.9 (SD 3.3) Range 6–18	12.8 (SD 1.7) Range 10–15	12.4 (SD 1.7) Range 10–15
Male (%)	83.2	53.6	49.7	87.6	50	49.6	49.8	45.2
Inattentive ADHD rating score (mean)	21.2 (SD 5.4)	4.4 (SD 4.8)	Not available	21.1 (SD 5.5)	7.8 (SD 7.8)	4.1 (SD 4.7)	Not available	7.5 (SD 7.7)
Hyperactive ADHD rating score (mean)	20.0 (SD 6.3)	2.9 (SD 4.4)	Not available	19.7 (SD 6.4)	7.0 (SD 7.7)	2.7 (SD 4.4)	Not available	5.8 (SD 7.1)
Primary carer has further or higher education (%)	28.2	74.0	33.0	27.7	29.1	80.1	33.0	32.6
Employment deprivation^a^ (%)	10.7	8.2	10.6	10.1	10.5	8.2	10.0	10.5
Income deprivation^b^ (%)	17.1	11.4	17.5	16.8	17.1	11.4	17.7	16.7
Primary carer cohabiting with partner (%)	67.4	87.8	72.9	65.4	72.4	91.2	76.1	72.5
Primary carer not employed (%)	58.2	11.7	35.7	59.9	55.3	11.8	38.0	59.1
Primary carer positive ADHD screen	36.4 % (*n* = 459)	6.4 % (*n* = 187)	Not available	35.7 % (*n* = 297)	37.3 % (*n* = 327)	6.1 % (*n* = 132)	Not available	38.4 % (*n* = 172)
Secondary carer positive ADHD screen	36.7 % (*n* = 210)	9.0 % (*n* = 134)	Not available	31.2 % (*n* = 128)	32.9 % (*n* = 167)	7.2 % (*n* = 97)	Not available	31.8 % (*n* = 88)

^a^Proportion of working age population in LSOA or data zone (for Scotland) that are employment deprived

^b^Proportion of population in LSOA or data zone that are income deprived

There were two independent variables of interest: having a diagnosis of ADHD and having a sibling with a diagnosis of ADHD. The regressions controlled for a broad range of child and household characteristics (age, gender, the number of children in the household, education level of carers, and employment and income deprivation within the local area^5^ and an adult ADHD screen). We ran the models with and without controlling for the primary carers ADHD screen to see how this affected results. We also ran the models with and without parental relationship and employment status. Since these could have been caused, in part, by living with a child with ADHD [39] controlling for these family level factors may produce an underestimate of the full impact of childhood ADHD. For siblings we also included their own ADHD symptoms through including the hyperactivity sub-score and the conduct problems sub-score of the SDQ as an additional control. Siblings without a diagnosis of ADHD may still experience some ADHD symptoms hence any deficit in health or well-being could have arisen due to their own ADHD symptoms rather than as a consequence of living with a sibling with ADHD. We show our regressions with and without the inclusion of the sibling SDQ sub-scores as it is also possible that rather than being caused by the siblings own-ADHD this behaviour could be causally related to the presence of a sibling with ADHD, for example, through copying older siblings, or reduced parenting time for the non-ADHD siblings, or arise from the general level of family disruption.

Each child outcome measure (CHU-9D, EQ-5D-Y profile, happiness with life and family scores, hours of sleep, sibling bullying) is treated as a dependent variable and modeled as a function of child and family characteristics. We adopt a model that is suitable to each outcome measure in question, with consideration given to the nature and distribution of the outcome measure. Life and family happiness, and hours of sleep are treated as cardinal and analysed using linear models (OLS). Responses to bullying questions and EQ-5D-Y are analysed using ordered logit models. The bounded nature of the CHU-9D utility instruments which cannot go above one at full health and the positive skew of the data with many values at full health make it suitable for analysis with the tobit model [40]. Robust standard errors are used, clustered at the household level to account for households with more than one child with ADHD or more than one eligible sibling.

## Results

### Study population

The background characteristics of the children with ADHD, their siblings and the respective controls are shown in Table 1. The ADHD-family group contains a greater percentage of boys. The SYC-control group contained primary carers with greater education attainment, who were more likely to be employed, in a cohabiting relationship and less likely to have a positive ADHD screen. These differences were less stark between the USoc-control and the ADHD-family group.

These differences in initial carer characteristics, particularly between SYC-control and the ADHD-family group, highlight the importance of the matching procedure and the need to include covariates within the regression models. The matching process prunes or drops cases were a good match cannot be found and then applies weights to the control group to create a new weighted-control group which is more similar to the ADHD-family group in terms of covariates. For the SYC comparison this resulted in dropping 37 ADHD family group children and 12 SYC children, leaving a sample of 184 SYC and 439 ADHD-family group children. For the USoc comparison of just the 10–15-year olds, the matching resulted in dropping 730 of the 4,234 USoc children and none of the ADHD-family group leaving a sample of 3,504 and 307, respectively. For the SYC comparison of siblings the matching resulted in dropping 39 ADHD family group children and 3 from the SYC leaving a sample size of 298 and 133, respectively. For the USoc comparison the matching resulted in dropping 271 of the 3,477 children from the USoc and 1 of the 178 children from SYC leaving a sample of 3,206 and 177, respectively.

Table 2 summarise the outcome measures for the children with ADHD, siblings and their controls (further details are shown in the on-line supplement Table S.1).

**Table 2:** Descriptive data on the children with ADHD, siblings and their controls, prior to matching

	Children with ADHD and their controls				Siblings and their controls			
	ADHD-family group diagnosed children (*N* = 476)	SYC (*N* = 196)	USoc (10–15 years) (*N* = 4,234)	ADHD-family group diagnosed children (10–15 years) (*N* = 307)	ADHD-family group siblings (*N* = 337)	SYC (those with appropriate aged siblings) (*N* = 136)	USoc (those with appropriately aged siblings) (*N* = 3,477)	ADHD family group siblings (10–15 years) (*N* = 178)
EQ-VAS (0–100)	80.16 (SD 20.61) Range 0–100 *n* = 456	86.93 (SD 14.71) Range 30–100 *n* = 193	NA	79.75 (SD 19.10) Range 12–100 *n* = 291	83.66 (SD 16.60) Range 5–100 *n* = 291	87.74 (SD 13.65) Range 35–100 *n* = 133	NA	82.70 (SD 15.62) Range 29–100 *n* = 158
CHU-9 (0–1)	0.82 (SD 0.12) Range 0.33–1 *n* = 419	0.88 (SD 0.09) Range 0.39–1) *n* = 189	NA	0.82 (SD 0.11) Range 0.45–1 *n* = 271	0.86 (SD 0.10) Range 0.35–1 *n* = 268	0.88 (SD 0.10) Range 0.39–1 *n* = 131	NA	0.85 (SD 0.10) Range 0.35–1 *n* = 148
Happiness with life (1–7)	5.38 (SD 1.59) *n* = 459	5.88 (SD 1.13) *n* = 193	5.88 (SD 1.18) *n* = 4,199	5.34 (SD 1.50) *n* = 294	5.40 (SD 1.51) *n* = 290	5.86 (SD 1.13) *n* = 133	6.38 (SD 1.03) *n* = 3,457	5.32 (SD 1.45) *n* = 156
Happiness with family (1–7)	5.98 (SD 1.20) *n* = 460	6.39 (SD 0.91) *n* = 193	6.37 (SD 1.05) *n* = 4,212	5.89 (SD 1.18) *n* = 295	5.76 (SD 1.34) *n* = 291	6.41 (SD 0.91) *n* = 133	6.38 (SD 1.03) *n* = 3,457	5.78 (SD 1.29) *n* = 157
Hours of sleep per day	8.62 (SD 1.65) Range 3.0–12.0 *n* = 327	9.61 (SD 1.09) Range 7.0–12.0 *n* = 141	NA	8.50 (SD 1.58) Range 3–12 *n* = 209	NA	NA	NA	NA

### Children with ADHD diagnosis

When compared with SYC controls, children diagnosed with ADHD have significantly poorer HRQoL across all measures (Table 3). We found utility scores (column 1 of Table 3) to be 0.063 lower for the CHU-9D and 6.93 lower for the EQ-VAS. When the primary carer ADHD screen was added to the model (column 2) these deficits remained similar. Including primary carer cohabitation and employment status (column 3) they fell slightly (to −0.57 for the CHU-9D and −5.806 for the EQ-VAS) but remained strongly significant. Including the secondary carer ADHD screen (column 4) increased the magnitude of effect CHU-9D but resulted in a loss of significance for the EQ-VAS, and gave a considerably smaller sample size.

**Table 3:** Marginal effects on health and well-being outcomes

Variables	SYC control			
	(1) Standard controls	(2) As (1) plus primary carer ADHD screen	(3) As (2) plus relationship and job status of primary carer	(4) As (3) plus secondary carer ADHD screen
EQ-VAS				
ADHD	−6.930***	−6.301**	−5.806**	−3.883
PC possible ADHD		0.349	0.318	−2.882
PC partner at home			−2.390	18.795
PC no job			−4.588**	−1.831
SC possible ADHD				−6.082*
*N*	602	578	573	315
Adj *R* ^2^	0.0591	0.0601	0.0692	0.0817
CHU-9				
ADHD	−0.063***	−0.061***	−0.057***	−0.075***
PC ADHD score		−0.006	−0.006	−0.013
PC partner at home			−0.013	−0.032
PC no job			−0.024	−0.004
SC possible ADHD				−0.021
*N*	569	546	541	298
Hours of sleep				
ADHD	−0.867***	−0.740***	−0.647***	−0.542**
PC possible ADHD		−0.445**	−0.361**	−0.469*
PC partner at home			0.440**	1.210**
PC no job			−0.086	−0.146
SC possible ADHD				−0.035
*N*	436	416	413	251
Adj *R* ^2^	0.202	0.207	0.221	0.216

Children with ADHD and matched controls from the SYC-control

We also considered predictors of individual item levels within the utility instruments, the CHU-9D and the EQ-5D-Y, using ordered logit models (see on-line supplement Table S.2 and S.3). This found that the children with ADHD reported significantly poorer outcomes in problems with school work, feeling annoyed, having had problems sleeping the previous night, daily routine, and joining in with activities, but not in feeling sad, feeling worried, being in pain or feeling tired. For the EQ-5D-Y items the children with ADHD report more problems with washing/dressing, usual activities, pain/discomfort and feeling worried/sad/unhappy, but no differences in mobility.

Children with ADHD report 52 min less sleep per night than their matched SYC-controls in the model with standard controls. Including the primary carer ADHD screen as a control reduces this to 44 min difference, and further including employment, and relationship status (column 3) reduces this further to about 39 min difference. Where the primary carer is cohabiting this results in the child getting 26 min more sleep per night compared with having a primary carer who is not in a co-habiting relationship. Those children with primary carers who screen positive for possible ADHD have about 22–27 min less sleep per night.

Comparisons to the SYC-control and to the USoc control (see Table 4) both find children with ADHD are less happy with their life overall; at least 0.5 on a 1–7 scale and robust to the inclusion of additional controls. Satisfaction with family is also significantly lower in both comparisons with the magnitude slightly greater in the USoc comparison (−0.344 for the SYC and −0.488 in the USoc, for the model with standard controls). Once primary carer employment status is included in the model only the USoc comparison remains significant.

**Table 4:** Marginal effects on subjective well-being outcomes

	SYC control group				USoc control group	
	(1) Standard controls	(2) As (1) plus primary carer ADHD screen	(3) As (2) plus relationship and job status of primary carer	(4) As (3) plus ADHD screen of secondary carer	(5) Standard controls	(6) As (5) plus relationship and job status of main carer
Happy with family						
ADHD	−0.344**	−0.294*	−0.252	−0.401***	−0.488***	−0.505***
PC poss ADHD		−0.090	−0.098	−0.145		
PC cohabiting			−0.157	−0.011		0.197***
PC no job			−0.273*	−0.200		0.091*
SC poss ADHD				0.016		
*N*	606	582	577	317	3,780	3,433
Adj *R* ^2^	0.0530	0.0524	0.0622	0.0857	0.0438	0.0533
Happy with life						
ADHD	−0.575***	−0.505**	−0.525***	−0.650**	−0.546***	−0.545***
PC poss ADHD		−0.201	−0.213	−0.488**		
PC cohabiting			−0.252	−0.368		0.205***
PC no job			−0.140	−0.033		0.045
SC poss ADHD				−0.058		
*N*	605	581	576	316	3,770	3,423
Adj *R* ^2^	0.0450	0.0400	0.0442	0.0657	0.0218	0.0282

Children with ADHD and matched controls from the USoc

The CHU-9D models use a weighted tobit and show average marginal effects. The other models use weighted OLS. Controls include: child’s age, gender, number of children in the household, % employment deprived in the area and % income deprived, primary carer having a further or higher education qualification, primary carer having A level or equivalent qualification, primary carer having some form of formal qualification below A level. A constant is also included. *** p < 0.01, ** p < 0.05, * p < 0.1, these are based on robust standard errors which are clustered at the household level. Full details of these regressions are available from the authors

There was a difference in reports of sibling bullying between the children with ADHD and children from both control groups. As the data is ordinal it was analysed using order logit models which estimates the differences in the probability of responding to each of the four response categories. Table 5 shows a summary of significant findings rather than each marginal effect for each response category. The USoc comparison finds significantly more sibling bullying reported (both to and by children with ADHD) across all four bullying questions. However, the SYC comparison finds only significantly more frequent incidence of children with ADHD taking their siblings belongings and, in only the model with standard controls, calling them names.

**Table 5:** Summary of the significance on differences in frequency of bullying; children with ADHD versus matched controls from SYC and USoc (taken from ordered logit models)

	South Yorkshire Cohort				Understanding Society	
	(1) Standard controls	(2) As (1) with primary carer ADHD control	(3) As (2) with relationship and job status of primary carer	(4) As (3) with secondary carer ADHD	(5) Standard controls	(6) As (5) plus relationship and job status of primary carer
Hit, kick or push you	×	×	×	×	√	√
Take your belongings	×	×	×	×	√	√
Call you nasty names	×	×	×	×	√	√
Make fun of you	×	×	×	×	√	√
Hit, kick or push them	×	×	×	×	√	√
Take their belongings	√	√	√	√	√	√
Call them nasty names	√	×	×	×	√	√
Make fun of them	×	×	×	×	√	√

### Siblings

Table 6 shows the impact of living with a sibling with ADHD. The matched comparisons find no impact of having a sibling with ADHD upon the HRQoL measures (CHU-9D, EQ-VAS), except for the CHU-9D for the most complete model. The siblings do, however, report substantially lower happiness with life overall (between 0.321 and 0.597 in the different models) and with their family (between 0.443 and 0.762) on a 1–7 scale, although this is not always significant in the smaller SYC comparison. The siblings express greater dissatisfaction with their family than the children with ADHD (for example, using the USoc comparison we see a deficit of 0.448 for the children with ADHD and, when controlling for own SDQ sub-scores, a deficit of 0.554 for siblings). Perhaps more surprising is that the reduction in their happiness with life overall is of a similar magnitude to the deficit of that for the children with ADHD.

**Table 6:** Marginal effects on health and well-being outcomes

Variables	South Yorkshire Cohort control				Understanding Society control		
	(1) Standard controls	(2) As (1) plus primary carer ADHD screen	(3) As (2) plus primary carer job and relationship status	(4) As (3) plus secondary carer ADHD screen	(5) Standard controls	(6) As (5) plus SDQ hyperactivity and conduct problems	(7) As (6) plus and primary carer relationship and job status
EQ-VAS							
Sibling with ADHD	−1.740	−0.928	−0.828	−2.820	NA	NA	NA
PC ADHD screen		1.511	1.296	1.048			
Own SDQ-hyperactive		−0.620	−0.491	−0.313			
Own SDQ-conduct		−1.620**	−1.783**	−2.172***			
PC partner at home			−3.439	NA			
PC no job			−3.591	−2.101			
SC ADHD screen				−5.277*			
*N*	388	365	362	221			
Adjusted *R* ^2^	0.0416	0.0981	0.108	0.154			
CHU-9D							
Sibling with ADHD	−0.008	−0.002	0.000	−0.035**	NA	NA	NA
PC ADHD screen		0.010	0.012	−0.004			
Own SDQ-hyperactive		−0.005*	−0.004	0.000			
Own SDQ-conduct		−0.010***	−0.011***	−0.018***			
PC partner at home			−0.011	NA			
PC no job			−0.024	0.006			
SC ADHD screen				−0.004			
*N*	366	343	340	210			
Happy with family							
Sibling with ADHD	−0.443	−0.499*	−0.455	−0.762***	−0.567***	−0.554***	−0.595***
PC ADHD screen		0.247	0.290	0.091			
Own SDQ-hyperactive		−0.038	−0.017	−0.010		−0.021**	−0.013
Own SDQ-conduct		−0.039	−0.062*	−0.164***		−0.131***	−0.133***
PC partner at home			−0.016				0.153***
PC no job			−0.238	0.254			0.176***
SC ADHD screen				0.280			
*N*	387	364	361	219	3,344	3,312	2,993
Adjusted *R* ^2^					0.056	0.119	0.125
Happy with life							
Sibling with ADHD	−0.461	−0.338	−0.321	−0.597**	−0.521***	−0.562***	−0.587***
PC ADHD screen		0.122	0.130	0.061			
Own SDQ-hyperactive		0.006	0.021	0.022		−0.070***	−0.070***
Own SDQ-conduct		−0.166***	−0.187***	−0.258***		−0.152***	−0.143***
PC partner at home			−0.234				0.114*
PC no job			−0.264	0.047			0.054
SC ADHD screen				0.337			
*N*	386	363	360	218	3,335	3,303	2,983
Adjusted *R* ^2^					0.0178	0.123	0.120

Siblings of children with ADHD and matched controls

The CHU-9D model uses a weighted tobit and show average marginal effects. The other models use weighted OLS. Controls include: child’s age, gender, number of children in the household, % employment deprived in the area and % income deprived, primary carer having a further or higher education qualification, primary carer having ‘A’ level or equivalent qualification, primary carer having some form of formal qualification below ‘A’ level. A constant is also included. ***p < 0.01, **p < 0.05, *p < 0.1, these are based on robust standard errors which are clustered at the household level. Full details of these regressions are available from the authors

Own conduct problems, as identified by parent completion of the SDQ conduct problem items, are negatively related to the EQ-VAS and the CHU-9D. When only the standard controls are included (column 2) we see that each additional point on the 0–10 scale lowers the CHU-9 by 0.010 and the VAS by 1.620. Both control groups find a strong negative impact of own conduct problems on happiness with life. Only the USoc comparison also finds a negative impact of hyperactivity symptoms. The USoc comparison also finds a significantly negative relationship between own conduct problems and happiness with family. However, the direction of causality is not clear in these relationships.

Table 7 shows a summary of the bullying comparisons for the siblings. The SYC comparison finds siblings of children with ADHD report increased frequency of their siblings calling them names and taking their belongings. However, these effects are no longer significant with the full set of controls. No significant difference is found in physical bullying. The siblings also report increased frequency of themselves calling their brother/sisters nasty names. The USoc comparison finds significantly more bullying across all questions, both to and from the siblings.

**Table 7:** Summary of the significance on differences in frequency of bullying; siblings of children with ADHD versus matched controls from SYC and USoc (taken from ordered logit models)

	SYC				USoc		
	(1)	(2)	(3)	(4)	(5)	(6)	(7)
	Standard controls	As (1) with primary carer ADHD screen and SDQ controls	As (2) with relationship and job status of primary carer	As (3) with secondary carer ADHD screen	Standard controls	As (5) with SDQ controls	As (6) and relationship and job status of primary carer
Hit, kick or push you	×	×	×	×	√	√	√
Take your belongings	√	√(10 %)	√(10 %)	√	√	√	√
Call you nasty names	√	×	×	×	√	√	√
Make fun of you	×	×	×	×	√	√	√
Hit, kick or push them	×	×	×	×	√	√	√
Take their belongings	×	×	×	×	√	√(10 %)	√
Call them nasty names	√	√	√	√	√	√	√
Make fun of them	√(10 %)	×	×	√(10 %)	√	√	√

## Discussion

After making careful adjustments to ensure an appropriate comparison, ADHD was found to be associated with a substantial reduction in the quality of life of patients. Patients, who were being treated for their ADHD, still experienced lower health, lower subjective well-being, less sleep and elevated bullying compared with children who did not have ADHD. This is consistent with previous studies that have identified a health and well-being loss from childhood ADHD [5–7, 9–14].

The utility scores for children with ADHD that are currently being treated are 0.06 (or half of a SD) lower based on the CHU-9D than children without ADHD. This difference is substantial relative to the typical minimum important difference in utility scores [41] and remained even after controlling for the primary carer’s own ADHD screen, employment and relationship status.

Children with ADHD give a poorer evaluation of their family and of their life overall (about a third of a SD) and clearly experience lower subjective well-being than children without ADHD. There is little evidence on the impact of other health conditions on children available to compare to these findings, but this magnitude of effect is strong relative to the impact of family circumstances [42].

Children with ADHD sleep significantly less than children without ADHD. Sleep deficit is one outcome in which our controls play an important role. The raw mean difference between the children with ADHD and their SYC-controls is about 1 h (see Table 1), but between 15 and 28 min of this difference can be accounted for by differences in carer characteristics (see Table 2). We found that having a primary carer with a positive ADHD screen resulted in children getting less sleep, and the primary carer having a partner living at home resulted in substantially more sleep. This suggests that single parents/carers, and parents/carers with ADHD symptoms themselves need greater support with sleep routines. Sleep problems may be attributed to both ADHD and to secondary low mood, hence potentially the impact of carer characteristics on sleep may be mediated via the child’s mood. Interestingly, despite reporting problems with their sleep within the CHU-9D, children with ADHD do not report feeling significantly more tired. However, this reduction in sleep could still be having other short and longer term consequences on the individual and on the sleep patterns of other members of the family.

Whilst no robust deficit is found for utility scores in children who live with a sibling with ADHD they were clearly identified as less happy with their family and less happy with their life overall. The siblings own ADHD symptoms, particularly conduct problems, are negatively related to many of the outcome measures, but interestingly controlling for own ADHD symptoms does not alter the magnitude of the decrement of living with a sibling diagnosed with ADHD on life satisfaction and family satisfaction. The magnitude of the dissatisfaction siblings have with their family and with life overall is surprisingly similar to that for the children with ADHD. This suggests both children with ADHD and their siblings have substantial unmet needs in terms of their overall happiness and well-being.

These low levels of reported happiness with their family, particularly from siblings, may be a result of sibling bullying. Both children with ADHD and their siblings report elevated levels of bullying compared to matched controls. The USoc comparison found that both children with ADHD and their siblings are both victims and victimizers of bullying, with all measures of bullying significantly higher than in the control group. The SYC comparison found that children with a sibling with ADHD reported a significantly higher frequency of their siblings taking their belongings, and also a significantly higher frequency of calling their siblings names. Children with ADHD corroborated this and reported a higher frequency of taking their siblings things. Bowes et al. [43] found that the frequency of sibling bullying at around age 12 was predictive of depression, anxiety and self-harm at 18. This suggests a need to consider interventions specifically targeted at addressing sibling bullying in families where a child has ADHD.

The mean value for the EQ-VAS was 83.66 for our sample of children with ADHD; considerably higher than the EQ-VAS proxy-score of 72.4 found by Secnik et al. [21]. Given that the two samples appear broadly similar (age 11.8 vs. 12.6, percentage boys 83 vs. 88, ADHD RS 41.2 vs. 37.2 for our data and Secnik et al. [21] sample, respectively), this may suggest that parents perceive the health state of children with ADHD as worse than the children perceive it themselves.

The CHU-9D utility decrement of childhood ADHD of around 6 % is very close to the difference in the EQ-VAS, which is also around 6 %. Whilst the two instruments do not share the same anchors (0 on the EQ-VAS being ‘worst possible health’ rather than ‘dead’), making direct comparison problematic, this does offer some support for validation for the use of the CHU-9D in terms of the magnitude of utility loss in children with ADHD within the current treatment provision. One of the problems with the CHU-9D is the high number of missing values in the CHU-9D for the item on schooling (9 % of children with ADHD with a complete EQ-5D-Y have missing values for CHU-9D) which may have arisen due to the child being currently excluded or not attending school and therefore not being clear how to respond to the question about ‘today’ if they did no school or homework ‘today’. This is a shame since if the child is currently not attending school, but they are of school age, then their school related problems are considerable. However, re-running the analysis with different assumptions made for this response makes minimal difference to our conclusion.^6^

### Strengths

A key strength of the study is the ability to control for a broad range of household characteristics and the carers’ ADHD screen to account for the clustering of ADHD across families. Surprisingly, adjustments made within the matching process and the regression analysis had only minimal impact upon the differences between the ADHD-family group and the control group. The raw mean difference in CHU-9D, for example, is about 6 % (see Table 1) and with all the appropriate adjustments the difference is also about 6 % (see Table 3). Similarly, for happiness with life the raw mean difference is about 0.5 on a 1–7 scale (see Table 1) and with all the appropriate adjustments the difference is also about 0.5 (see Table 3). This suggests that the health and well-being loss from having childhood ADHD is independent of individual and household characteristics. This is not, however, the case for understanding sleep patterns, which (as discussed above) are strongly influenced by other household characteristics.

### Limitations

The study has a number of limitations. First, the analysis is based on observational, cross-sectional data. We are therefore constrained in being able to imply too much about causality. Whilst the control groups are closely matched to the ADHD-family group in terms of observable characteristics, we cannot be certain that there are not differences in unobserved characteristics which have not been accounted for. Parenting style, such as use of criticism rather than praise, may be one such unobservable. A negative parenting style may exacerbate ADHD symptoms, which could result in a correlation between ADHD diagnosis and parental style. If a parenting style has implications for children’s health and well-being, it could be the parenting style which is driving any outcome differences. We have no way of controlling for initial parenting style, particularly given that current parenting style may be a response to the stresses of interacting with a child with ADHD [44].

Our results relate to children in the UK currently being treated for ADHD and does not include untreated ADHD cases, nor does it include patients with a current diagnosis of CD (although it is possible that some of our sample had CD which had not been formally diagnosed and children with less severe forms of oppositionality were included. While this in some way limits the generalisability of the findings it does make the interpretation of the effects more straightforward in that it removes the ambiguity introduced by having both ADHD and CD in the sample—conditions which may both impact on the everyday lives of the children.

It may be that the presence of comorbidities (such as opposition defiant disorder) drives the impact on health and quality of life of both the child with ADHD and their siblings. To explore this would require a consistent diagnosis of comorbidities across the sample which is not available here.

A further limitation with the study is that we do not have data on all siblings within the ADHD family group. There may be selection into participating in the study which could be related to general compliance of the child, or to those who feel aggrieved at living with a child with ADHD. However, most eligible siblings did complete the questionnaires (5 % of eligible siblings of families engaging with the study do not complete questionnaires and a further 5 % have key missing data required for the analysis).

The study unavoidably focuses the children on living with an ADHD or living with a sibling with ADHD. In answering questions such as happiness with their family siblings are likely to give greater attention to aspects of living with a sibling with ADHD than if they answered questions with no framing towards ADHD. Furthermore, children may have perceived an incentive to overstate their problems.

We rely on the assumption that children in the control groups do not have ADHD themselves or a sibling with ADHD (diagnosed or undiagnosed). For the SYC-control group the self-reported presence of a child with ADHD was an exclusion criteria. The USoc control group is a representative sample of a households in the UK, hence potentially includes families with children with ADHD. This is likely to result in a slight underestimation of the overall impact of having ADHD, or having a sibling with ADHD, but since the prevalence is likely to be around 4 % [2] this should have minimal impact upon the results.

## Conclusion

Children with ADHD, treated within the UK, experienced a lower level of HRQoL than children without ADHD. This was the case even after controlling for differences in household characteristics and a control to capture possible ADHD in parents/carers. Using the pediatric utility instrument, the CHU-9D, we find a decrement in utility of around 6 %. This implies a substantial unmet health need, despite receiving current treatment. Both children with ADHD and their siblings reported that they were substantially less happy with their family and less happy with life overall. Both groups also suffer elevated levels of intra-family bullying.

## Electronic supplementary material

Below is the link to the electronic supplementary material.

Supplementary Tables (DOCX 49 kb)

## Footnotes

1 The term ‘utility’ in this context is used in a very general sense as a reflection of value or how ‘good’ a state would be to live in. It is drawn either from direct judgement the individual (or their proxy) makes about their own quality of life, or through taking the average judgement from a sample of individuals about health states described to them.

2
http://clahrc-sy.nihr.ac.uk/south-yorkshire-cohort.html.

3 “The next few questions are about how you feel about your life. The faces express different types of feelings. Below each face is a number where ‘1’ is completely happy and ‘7’ is not at all happy. Please tick the box which comes closest to how you feel about each of the following things…your family/which best describes how you feel about your life as a whole?” (understanding society, youth questionnaire).

4 Up to two missing values were tolerated and the score calculated pro-rata.

5 The local level income and employment deprivation was derived from the 2010 proportion of the population income deprived according to benefit claims at the lower level super outcome area (LSOA) in England and at the slightly smaller data zone level for Scotland.

6 Assuming the second or third level of problems increases the marginal effect of having ADHD on the CHU-9D in the standard controls model from 0.063 to 0.064; assuming the fourth or fifth level of problems increases this to 0.065.
