# Supplementary material for: The impact of ADHD on the health and well-being of ADHD children and their siblings
Source: Eur Child Adolesc Psychiatry. 2016 Apr 1;25(11):1217–31. doi: 10.1007/s00787-016-0841-6 (PMC5083759; doi:10.1007/s00787-016-0841-6)
Supplement: Supplementary file 1 — Supplementary Tables (DOCX 49 kb) [file 787_2016_841_MOESM1_ESM.docx]

**Table S.1 Descriptive data for ADHD children and controls from SYC.**

| **Bullying** | Children with ADHD  with siblings  n=368 | SYCcontrols with siblings  n=153 |
| --- | --- | --- |
| Being bullied |  |  |
| Hit, kick or push you   - Never - Not much - Quite a lot - A lot - Missing | 66  103  63  118  18 | 35  52  30  33  3 |
| Take your belongings   - Never - Not much - Quite a lot - A lot - missing | 146  103  44  54  21 | 75  55  13  7  3 |
| Call you names   - Never - Not much - Quite a lot - A lot - missing | 82  84  57  124  21 | 51  56  26  17  3 |
| Make fun of you   - Never - Not much - Quite a lot - A lot - Missing | 129  73  54  90  22 | 59  49  19  23  3 |
|  |  |  |
| Bullying |  |  |
| Hit, kick or push them   - Never - Not much - Quite a lot - A lot - missing | 48  105  73  122  20 | 41  56  28  24  4 |
| Take their belongings   - Never - Not much - Quite a lot - A lot] - missing | 142  91  57  58  20 | 96  39  12  2  4 |
| Call them names   - Never - Not much - Quite a lot - A lot - missing | 70  92  81  106  19 | 61  50  25  12  5 |
| Make fun of them   - Never - Not much - Quite a lot - A lot - Missing | 111  84  67  87  19 | 63  48  25  13  4 |
|  |  |  |
| **EQ-5D-Y** | Children with ADHD  n=476 | SYC controls n=196 |
| Mobility*(walking about)*   - no problems walking about - some problems walking about - a lot of problems walking about - missing | 389  59  13  15 | 184  8  1  3 |
| Looking after myself *(washing/dressing)*   - no problems washing or dressing myself - some problems washing or dressing myself - a lot of problems washing or dressing myself - missing | 333  103  24  16 | 185  8  0  3 |
| Doing usual activities *(for example, going to school, hobbies, sports, playing, doing things with family or friends)*   - no problems doing my usual activities - some problems doing my usual activities - a lot of problems doing my usual activities - missing | 319  122  20  15 | 183  9  1  3 |
| Having pain or discomfort   - no pain or discomfort - some pain or discomfort - a lot of pain or discomfort - missing | 299  144  17  16 | 145  44  4  3 |
| Feeling worried, sad or unhappy   - not worried, sad or unhappy - a bit worried, sad or unhappy - very worried, sad or unhappy - missing | 315  129  18  14 | 145  46  2  3 |
|  |  |  |
| **CHU-9D** |  |  |
| **Worried**   - I don’t feel worried today - I feel a little bit worried today - I feel a bit worried today - I feel quite worried today - I feel very worried today - missing | 333  66  34  17  12  14 | 130  46  11  5  1  3 |
| **Sad**   - I don’t feel sad today - I feel a little bit sad today - I feel a bit sad today - I feel quite sad today - I feel very sad today - missing | 340  67  29  16  9  15 | 140  39  11  1  2  3 |
| **Pain**   - I don’t have any pain today - I have a little bit of pain today - I have a bit of pain today - I have quite a lot of pain today - I have a lot of pain today - missing | 306  90  34  16  13  17 | 129  47  10  5  2  3 |
| **Tired**   - I don’t feel tired today - I feel a little bit tired today - I feel a bit tired today - I feel quite tired today - I feel very tired today - missing | 158  133  75  34  61  15 | 56  71  28  30  8  3 |
| **Annoyed**   - I don’t feel annoyed today - I feel a little bit annoyed today - I feel a bit annoyed today - I feel quite annoyed today - I feel very annoyed today - missing | 266  92  51  32  21  14 | 129  42  13  4  4  4 |
| **School Work/Homework (such as reading, writing, doing lessons)**   - I have no problems with my schoolwork/homework today - I have a few problems with my schoolwork/homework today - I have some problems with my schoolwork/homework today - I have many problems with my schoolwork/homework today - I can’t do my schoolwork/homework today - missing | 166  99  70  51  42  48 | 119  54  11  5  1  6 |
| **Sleep**   - Last night I had no problems sleeping - Last night I had a few problems sleeping - Last night I had some problems sleeping - Last night I had many problems sleeping - Last night I couldn’t sleep at all - missing | 193  112  74  47  34  16 | 124  45  18  4  2  3 |
| **Daily routine (things like eating, having a bath/shower, getting dressed)**   - I have no problems with my daily routine today - I have a few problems with my daily routine today - I have some problems with my daily routine today - I have many problems with my daily routine today - I can’t do my daily routine today - missing | 243  114  61  28  14  16 | 167  20  4  2  0  3 |
| **Able to join in activities (things like playing out with your friends, doing sports, joining in things)**   - I can join in with any activities today - I can join in with most activities today - I can join in with some activities today - I can join in with a few activities today - I can join in with no activities today - missing | 252  88  46  39  34  17 | 145  29  11  4  4  3 |

**Table S.2Marginal effect of being in the ADHD children-group versus controls from SYC for each domain of the CHU-9D (with standard controls)**

|  | Level 1 (no problems) | Level 2 | Level 3 | Level 4 | Level 5 |
| --- | --- | --- | --- | --- | --- |
| Worried | -0.0105 | 0.0050 | 0.0031 | 0.0015 | 0.0009 |
| Sad | 0.0605 | -0.0323 | -0.0163 | -0.0073 | -0.0047 |
| Pain | -0.1038 | 0.0519 | 0.0263 | 0.0136 | 0.0120 |
| Tired | -0.0843 | -0.0012 | 0.0220 | 0.0288 | 0.0347 |
| Annoyed | -0.1661* | 0.0492 | 0.0598** | 0.0345* | 0.0225 |
| School work | -0.2447*** | 0.0353** | 0.0650*** | 0.0758*** | 0.0686*** |
| Sleep | -0.2986*** | 0.0562*** | 0.0980*** | 0.0791*** | 0.0652*** |
| Daily routine | -0.3321*** | 0.1203*** | 0.1148*** | 0.0657*** | 0.0312* |
| Able to join in activities | -0.1989** | 0.0493* | 0.0458** | 0.0509** | 0.0528** |

**Table S.3Marginal effect of being in the ADHD children-group versus controls from SYC for each domain of the EQ-5D-Y (with standard controls)**

|  | Level 1 (no problems) | Level 2 | Level 3 |
| --- | --- | --- | --- |
| Mobility *(walking about)* | -0.0891 | 0.0717 | 0.0174 |
| Looking after myself *(washing/dressing)* | -0.2376** | 0.1789*** | 0.0586* |
| Doing usual activities | -0.3213*** | 0.2645*** | 0.0567** |
| Having pain or discomfort | -0.2076** | 0.1760** | 0.0316** |
| Feeling worried, sad or unhappy | -0.1407* | 0.1209* | 0.0198 |

Notes: Models in S.2 and S.3 use a weighted ordered logit and show average marginal effects. Controls include: child’s age, gender, number of children in the household, % employment deprived in the area and % income deprived, primary carer having a further or higher education qualification, primary carer having ‘A’ level or equivalent qualification, primary carer having some form of formal qualification below ‘A’ level. A constant is also included. *** p<0.01, ** p<0.05, * p<0.1, these are based on robust standard errors which are clustered at the household level. Full details of these regressions are available from the authors.
